# Detection of small (≤ 2 cm) pancreatic adenocarcinoma and surrounding parenchyma: correlations between enhancement patterns at triphasic MDCT and histologic features

**DOI:** 10.1186/1471-230X-14-16

**Published:** 2014-01-21

**Authors:** Michele Scialpi, Lucio Cagini, Luisa Pierotti, Francesco De Santis, Teresa Pusiol, Irene Piscioli, Michelle Magli, Alfredo D’Andrea, Luca Brunese, Antonio Rotondo

**Affiliations:** 1 Department of Surgical, Radiological and Odontostomatological Sciences, Division of Radiology 2, Perugia University, S. Maria della Misericordia Hospital, S. Andrea delle Fratte, Perugia 06134, Italy; 2 Department of Surgical, Radiological and Odontostomatological Sciences, Thoracic Surgery, Perugia University, S. Maria della Misericordia Hospital, S. Andrea delle Fratte, Perugia, Italy; 3 Complex Structure of General Surgery 2, Perugia University, S. Maria della Misericordia Hospital, S. Andrea delle Fratte, Perugia, Italy; 4 Department of Pathology, S. Maria del Carmine Rovereto Hospital, Rovereto, Italy; 5 Division of Radiology, Budrio Hospital, ASL Budrio, Bologna, Italy; 6 Department of Radiology, University Hospital of Parma, Parma, Italy; 7 Division of Radiology, San Giuseppe Moscati Hospital, Aversa, Caserta, Italy; 8 Department of Radiology, University of Molise, C.da Tappino, Campobasso, Italy; 9 Department of Experimental and Clinical Internistic, F. Magrassi- A. Lanzara, Second University of Naples, Naples, Italy

**Keywords:** Pancreas, Pancreas, Neoplasms, Helical Computed Tomography (CT), Triphasic Helical CT, Quantitative analysis

## Abstract

**Background:**

The aim is to assess the time-density curves (TDCs) and correlate the histologic results for small (≤ 2 cm) PDA and surrounding parenchyma at triphasic Multidetector-row CT (MDCT).

**Methods:**

Triphasic MDCT scans of 38 consecutive patients who underwent surgery for a small PDA were retrospectively reviewed. The TDCs were analyzed and compared with histologic examination of the PDA and pancreas upstream/downstream in all cases. Three enhancement patterns were identified: 1) enhancement peak during pancreatic parenchymal phase (PPP) followed by a rapid decline on portal venous phase (PVP) and delayed phase (DP) at 5 minutes (type 1 pattern: normal pancreas); 2) maximum enhancement in PVP that gradually decreases in DP (type 2 pattern: mild chronic pancreatitis or PDA with mild fibrous stroma); 3) progressive enhancement with maximum peak in DP (type 3 pattern: severe chronic pancreatitis or PDA with severe fibrous stroma). A p value less than 0.05 was considered statistically significant. Sensitivity was calculated for PDA detection and an attenuation difference with the surrounding tissue of at least 10 HU was considered.

**Results:**

PDA showed type 2 pattern in 5/38 cases (13.2%) and type 3 pattern in 33/38 cases (86,8%). Pancreas upstream to the tumor had type 2 pattern in 20/38 cases (52,6%) and type 3 pattern in 18/38 cases (47,4%). Pancreas downstream to the tumor had type 1 pattern in 19/25 cases (76%) and type 2 pattern in 6/25 cases (24%). Attenuation difference between tumor and parenchyma upstream was higher of 10 UH on PPP in 31/38 patients (sensitivity = 81.6%), on PVP in 29/38 (sensitivity = 76.3%) and on DP in 17/38 (sensitivity = 44.7%). Attenuation difference between tumor and parenchyma downstream was higher of 10 UH on PPP in 25/25 patients (sensitivity = 100%), on PVP in 22/25 (sensitivity = 88%) and on DP in 20/25 (sensitivity = 80%). Small PDAs were isodense to the pancreas upstream to the tumor, and therefore unrecognizable, in 8 cases (8/38; 21%) at qualitative analysis and in 4 cases (4/38; 10,5%) at quantitative analysis.

**Conclusions:**

The quantitative analysis increases the sensitivity for detection of small PDA at triphasic MDCT.

## Background

Detection of pancreatic ductal adenocarcinoma (PDA) by imaging is essential for an accurate tumor staging and for choosing an adequate treatment [1].

Although current Multidetector-row Computed Tomography (MDCT) protocols [2-6] maximize the attenuation differences between the hypovascular tumor and the surrounding parenchyma, about 10% of PDA is isoattenuating to the pancreatic parenchyma underlying [4-9], making diagnosis more difficult; in these cases to detect the PDA, the secondary signs (e.g. main pancreatic duct dilatation or the interrupted duct sign) can be extremely useful [7,8].

Histopathological characteristics of tumor and surrounding parenchyma represent the causes of misdiagnosis owing to obscurity of the lesion on a CT scan [5,8,10,11]. A similar degree of fibrosis in the tumor and surrounding pancreatic parenchyma, resulting in the form of mild or severe chronic obstructive pancreatitis, may determine a similar enhancement on MDCT precluding the identification of PDA.

The knowledge by triphasic MDCT of the different enhancement patterns of PDA, pancreatic parenchyma upstream (toward the tail) and downstream (toward the head) to the tumor and histopathological features related are essential to increase the sensitivity of MDCT in the detection of PDA.

Our aim is to correlate the time-density curves (TDCs) at triphasic MDCT with histological characteristics for small (≤ 2 cm) PDA and surrounding parenchyma.

## Methods

### Patient selection

From a retrospective review of pathological reports and surgical records we identified 127 patients with PDA who underwent pancreaticoduodenectomy, distal pancreatectomy or total pancreatectomy at our institution between January 2006 and March 2012.

Tumors without histological examination of surrounding tissue or greater than 2 cm, tumors with atrophy of the upstream pancreas (n = 73) and patients with incomplete or inadequate helical Computed Tomography (CT) protocol (n = 16) were excluded from the study.

The final study population consists of 38 patients (26 males and 12 females, mean age 67.6 years, range age 54–88 years) who underwent an identical triphasic MDCT protocol, ultrasonography and/or Magnetic Resonance imaging and in whom the histological and MDCT examination of PDA and pancreas upstream/downstream to the tumor was performed.

For this retrospective study the evaluation of data obtained in the clinical routine were followed and informed consent was obtained in all patients. Information gathered on this population was performed in compliance with the Declaration of Helsinki principles.

### Histological evaluation

The surgical specimens of the pancreaticoduodenectomy, distal pancreatectomy or total pancreatectomy were examined for the PDA (n = 38) and pancreatic parenchyma upstream (n = 38) and downstream (n = 25) to the tumor. The surgical specimens for pancreatic parenchyma downstream to the tumor were not available in 13 patients because, in these patients, the PDA was localized in the uncinate process.

PDA histologically showed high cellularity of tumor cells with dense mild or abundant fibrosis, mucin and/or necrosis.

Mild chronic pancreatitis histologically showed a limited involvement of pancreatic parenchyma upstream and downstream to the tumor corresponding to fibrosis, dilatation of small ducts and occasional presence of inflammatory cells.

Severe chronic pancreatitis histologically showed diffuse fibrosis, low presence of inflammatory cells, loss of acini and dilatation of the portion of the Wirsung’s duct in the upstream pancreatic parenchyma to the tumor.

### Multislice CT protocol

CT scans were performed with 16 slice (Light Speed Plus and Light Speed Pro 16, GE Healthcare, Milwaukee, USA) and 64 slice (Philips Brilliance, Nederlands, United Kingdom, and Light Speed Pro 64, GE Healthcare, Milwaukee, USA) MDCT. The injected vein was initially tested with 30 mL of saline solution to ensure proper function. Before the examination, 600–900 ml of water per os, in order to improve the visualization of the relationships between pancreatic head and duodenum, were given.

We use bolus tracking, positioning a circular region of interest (ROI) on the abdominal aorta at the level of the celiac axis and using an enhancement threshold of 150 Hounsfield Unit (HU). After a scout-view of the entire abdomen and an unenhanced CT phase of the upper abdomen, triple phase acquisition includes a pancreatic parenchymal phase (PPP) (started 25 seconds after threshold of 150 HU aortic enhancement), a portal venous phase (PVP) (started 40 seconds after the end of PPP acquisition) and a delayed phase (DP) (after 5-minutes to the start of injection). Nonionic iodinated contrast agent (Iopamidolo, Iopamiro 370 mgI/ml; Bracco, Milan, Italy and Iopromide, Ultravist 370 mgI/ml Schering AG, Berlin, Germany) is injected in a quantity ranged from 120 ml to 150 ml at a rate of 4 mL/sec from an antecubital vein using a 18 gauge needle.

Slice thickness was 2.5 mm, gantry rotation speed of 0.75 seconds, reconstruction index 1.25; beam pitch: 0,935:1; 120 kilovolts peak (kVp) and automatic tube current (milliampere; mA) modulation using z-axis (Auto mA technique) or dose modulation (Z-DOM).

MDCT examinations were completed with sagittal, coronal or curved multiplanar reconstructions (MPR).

### Images analysis

Image analysis had two steps: qualitative analysis and quantitative analysis of PDA and pancreatic parenchyma up/downstream to the tumor.

The qualitative and quantitative analysis were performed on a workstation for reporting and image processing (Advantage Workstation 4.2 GE Healthcare, Milwaukee, USA and MagicView, Philips Medical Systems, Best, Netherlands).

For the qualitative analysis of PDA, all images were retrospectively assessed by two radiologists with more than 20 years of experience in abdominal CT interpretation and the findings were made in consensus. The attenuation of PDA and pancreatic parenchyma up/downstream to the tumor was classified as hyperattenuation, isoattenuation and hypoattenuation on each phase of CT scan. Ancillary findings in diagnosis of pancreatitis and in detection of tumor were: distortion of the outlines of the pancreas, margins and homogeneity of tumor, dilatation of main pancreatic duct upstream to the tumor and of extrahepatic bile ducts.

The regions of interest (ROIs) were determined by one of the authors for PDA and pancreas upstream/downstream to the tumor. The quantitative analysis of PDA was performed to determine the tumor-to-surrounding pancreas attenuation difference, defined as the difference in attenuation between the tumor and the pancreas upstream or downstream. Attenuation values were obtained in a circular ROI ranged in size from 0.2 to 1 cm^2^. The ROI tumor values in the PPP, PVP and DP were measured in at least half of the mass from the portion of the tumor that had the greatest attenuation on the arterial phase images.

A tumor ROI of approximately 0.5 cm^2^ (range, 0.32–0.56 cm^2^) was maintained. Measurement of the attenuation in the pancreatic parenchyma up/downstream to the tumor was obtained at 1 cm from the margins of the tumor and excluded visible vessels, pancreatic ducts, cystic areas, calcifications or necrosis. A constant ROI area of parenchyma up-/downstream of approximately 1.0 cm^2^ (range, 0.5–1.0 cm^2^) was maintained. At least three measurements were performed in each tumor and in the up-/downstream parenchyma and the results were averaged.

A difference of more than 10 HU in mean attenuation between the tumor and the *pancreatic parenchyma upstream and/or downstream* was considered meaningful [4]. The difference in attenuation between each tumor and surrounding parenchyma was calculated as follow:

Attenuation difference = *mean tumor attenuation values* - *mean pancreas upstream and/or downstream attenuation values.*

A positive difference of enhancement indicates that the lesion is hyperdense compared with surrounding parenchyma and vice versa. When the difference in attenuation between the lesion and pancreatic parenchyma upstream or downstream to the lesion presents values <10 HU, the lesions are unrecognizable (PDA so called “isodense”).

After the assessment of PDA and pancreatic parenchyma up-/downstream to the tumor attenuation, the time-density curves (TDCs) of PDA and up-/downstream pancreatic parenchyma in each patient were generated and categorized into three patterns:

•* Type 1 pattern:* rapid rise to a peak on PPP followed by a rapid decline;

•* Type 2 pattern:* slow rise to a peak on PVP followed by a slow decline;

•* Type 3 pattern:* a progressive enhancement with peak on DP.

The patterns of TDCs from triphasic CT measured at the PDA, pancreas upstream and downstream were then compared with the corresponding histological pancreatic sections in each patient.

### Statistical analysis

For comparative analysis of the average values of attenuation (mean values in HU ± standard deviation [SD]) of PDA, parenchyma upstream and downstream was used Student t test. A p value less than 0.05 was considered statistically significant. Sensitivity was calculated for the identification of PDA compared with upstream and downstream pancreas in each CT phase. For the statistical analysis has been used a version of SPSS for Windows software (release13.0, SPSS Chicago, III).

## Results

The PDAs ranged in size (maximum diameter on the axial plane) from 1.2 to 2.0 cm (mean, 1.85 cm). PDA was located at the head (n = 17), uncinate process (n = 7), neck (n = 6) and body (n = 8) of the pancreas. Dilatation of main pancreatic duct (diameter ranged from 4 to 14 mm, mean 6.9 mm) was detected in 23 of 38 patients (60.5%).

The results of tumor appearance (hyper-, iso- and hypodense) at qualitative analysis in each CT phase with respect to pancreas upstream (n = 38) and downstream (n = 25) are shown in Table 1.

**Table 1:** CT attenuation patterns of tumor, pancreas upstream and pancreas downstream to the tumor at qualitative analysis by triphasic CT

	Pancreas upstream to the tumor (n = 38)*			Pancreas downstream to the tumor (n = 25)**		
**CT pattern of tumor attenuation**	**Hyperdense**	**Hypodense**	**Isodense**	**Hyperdense**	**Hypodense**	**Isodense**
**PPP**	8 (21.1)	20 (52.6)	10 (26.3)	-	25 (100)	-
**PVP**	4 (10.3)	22 (57.9)	12 (31.6)	-	22 (88)	3 (12)
**DP**	10 (26.3)	6 (15.8)	22 (57.9)	6 (24)	14 (56)	5 (20)

PPP: pancreatic parenchymal phase; PVP = portal venous phase; DP = delayed phase. *CT/histologic assessment in 38/38 patients; **CT/histologic assessment in 25/38 patients. Number in brackets indicates the percentage.

At qualitative analysis the PDA was isodense to the pancreas upstream to the tumor on PPP in 10/38 (26.3%), on PVP in 12/38 (31.6%) and on DP in 22/38 cases (57.9%). On qualitative triphasic CT analysis, small PDAs were isodense to the pancreas upstream to the tumor in 8 cases (8/38; 21%); on DP were detected 2 PDAs isoattenuating on PPP and PVP. The PDA was hypodense to the pancreas downstream to the tumor in 25/25 (100%) cases on PPP, hypodense and isodense on PVP in 22/25 (88%) and in 3/25 (12%) respectively and hypodense and hyperdense on DP in 14/25 (56%) and in 6/25 cases (24%) respectively.

The results of tumor appearance (hyper-, iso- and hypodense) at quantitative analysis in each CT phase with respect to pancreas upstream (n = 38) and downstream (n = 25) is shown in Table 2.

**Table 2:** CT attenuation patterns of tumor, pancreas upstream and pancreas downstream to the tumor at quantitative analysis by triphasic CT

	Pancreas upstream to the tumor (n = 38)*			Pancreas downstream to the tumor (n = 25)**		
**CT pattern of tumor attenuation**	**Hyperdense**	**Hypodense**	**Isodense**	**Hyperdense**	**Hypodense**	**Isodense**
**PPP**	9 (23.7)	22 (57.9)	7 (18.4)	-	25 (100)	-
**PVP**	5 (13.1)	24 (63.2)	9 (23.7)	-	22 (88)	3 (12)
**DP**	10 (26.3)	7 (18.4)	21 (55.3)	6 (24)	14 (56)	5 (20)

PPP: pancreatic parenchymal phase; PVP = portal venous phase; DP = delayed phase. *CT/histologic assessment in 38/38 patients; **CT/histologic assessment in 25/38 patients. Number in brackets indicates the percentage.

On quantitative triphasic CT analysis, small PDAs were isodense to the pancreas upstream to the tumor, and therefore unrecognizable, in 4 cases (4/38; 10,5%).

The mean attenuation values (HU ± SD) of PDA, pancreatic parenchyma upstream and downstream are shown in Table 3 and Additional file 1.

**Table 3:** Attenuation values (HU) related to diagram 1, of tumor, pancreas upstream and pancreas downstream to the tumor at triphasic CT

	**Pre-C**	**PPP**	**PVP**	**DP**
**Tumor**	37 ± 9.7	64 ± 22.6	82 ± 26.1	89.6 ± 23.2
**Pancreas upstream**	22.2 ± 12.2	83 ± 27	106 ± 23.7	100.8 ± 23.4
**Pancreas downstream**	38.8 ± 11.1	121 ± 35.5	110.4 ± 19.7	91.9 ± 16.1

Pre-C. pre-contrast; PPP: pancreatic parenchymal phase; PVP = portal venous phase;

DP = delayed phase.

Tumor shows progressive enhancement throughout the three phases with maximum peak in DP; pancreatic parenchyma upstream to the tumor shows maximum enhancement in PVP that gradually decreases in DP; pancreatic parenchyma downstream to the tumor shows maximum enhancement peak during PPP followed by a rapid decline on PVP and DP.

The mean attenuation values (HU ± SD) of pancreas upstream to the tumor were significantly higher than those of PDA on PPP, PVP and DP (p < 0.05) whereas the mean attenuation values of parenchyma downstream to the tumor were significantly higher than those of tumor in PPP and PVP (p < 0.05) but not significantly different in DP (p > 0.05).

The comparisons of the differences of mean attenuation values between PDA and pancreas upstream and downstream to the tumor on PPP, PVP and DP are shown in Additional file 2.

The difference in attenuation between tumor and parenchyma upstream was at least 10 HU on PPP in 31/38 patients (sensitivity = 81.6%), on PVP in 29/38 (sensitivity = 76.3%) and on DP in 17/38 (sensitivity = 44.7%). The difference in attenuation between tumor and parenchyma downstream was at least 10 HU on PPP in 25/25 patients (sensitivity = 100%), on PVP in 22/25 (sensitivity = 88%) and on DP in 20/25 (sensitivity = 80%).

Diagnostic indices of CT sensitivity in PDA detection on PPP, PVP and DP are summarized in Table 4. The quantitative analysis by triphasic CT increases the results of qualitative analysis in PDA detection with a sensitivity of 100% in PDA detection when pancreas downstream is considered.

**Table 4:** Diagnostic indices of triphasic CT sensitivity for detection of pancreatic ductal adenocarcinoma

	**PPP**	**PVP**	**DP**
Tumor vs upstream (n = 38)*	31/38 (81.6)	29/38 (76.3)	17/38 (44.7)
Tumor vs downstream (n = 25)**	25/25 (100)	22/25 ( 88)	20/25 (80)

PPP: pancreatic parenchymal phase; PVP = portal venous phase; DP = delayed phase. *CT/histologic assessment in 38/38 patients; **CT/histologic assessment in 25/38 patients. Number in the brackets indicates the percentage.

According to the time-density curves (TDCs) of PDA and pancreatic parenchyma upstream and downstream, type 1 pattern (enhancement peak during PPP followed by a rapid decline on PVP and DP) was revealed only in pancreas downstream in 19/25 cases (76%), type 2 pattern (a maximum enhancement in PVP that gradually decreases in DP) was revealed in PDA in 5/38 cases (13,2%), in pancreas upstream in 20/38 cases (52,6%) and in pancreas downstream in 6/25 cases (24%), while type 3 pattern enhancement (a progressive enhancement throughout the three phases with maximum peak in DP) was revealed in PDA in 33/38 cases (86,8%) and in pancreas upstream in 18/38 cases (47,4%).

The correlation between histopathological characteristics and TDCs for PDA, pancreas upstream and downstream shows that type 1 pattern corresponds to normal pancreas, type 2 pattern corresponds to mild chronic pancreatitis or PDA with mild fibrous stroma and type 3 pattern corresponds to severe chronic pancreatitis or PDA with abundant fibrous stroma.

**Figure 1:**
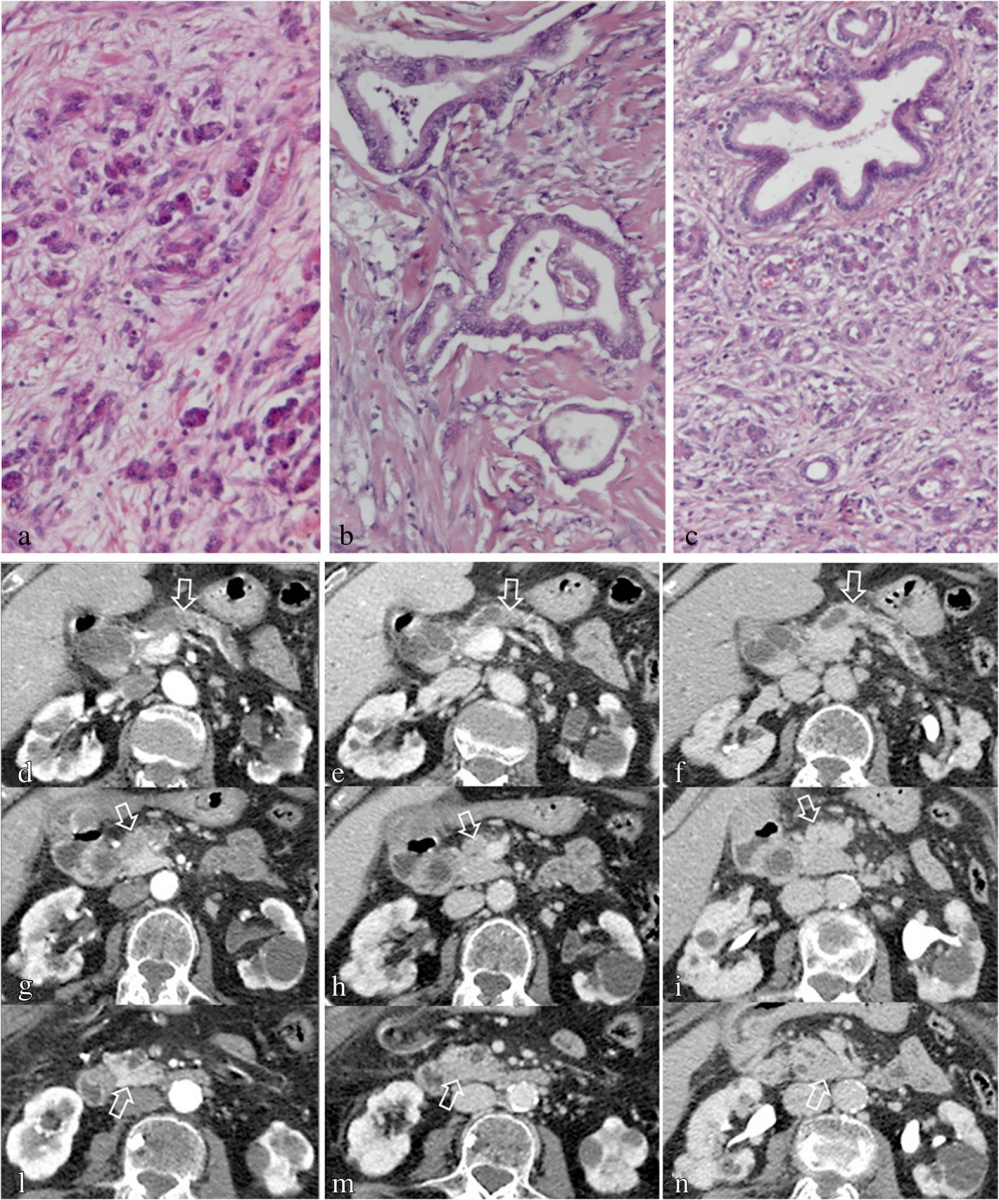
**Pancreatic ductal adenocarcinoma of the neck of the pancreas: histology with triple-phase helical CT correlation in severe and mild chronic pancreatitis of pancreas upstream and downstream respectively.** Severe diffuse fibrosis of upstream pancreatic tissue (HδE 100X) **(a)**, ductal pancreatic adenocarcinoma with intense severe desmoplastic stromal reaction consisting of dense collagen (HδE 100X) **(b)**, and extensive inflammatory infiltrate with mild fibrosis in downstream pancreas (HδE 100X) **(c)** and correspective pattern of progressive enhancement with maximum peak on delayed phase of the upstream pancreatic tissue (arrow in **d, e, f**), pancreatic ductal carcinoma (arrow in **g, h, i**) and maximum enhancement in pancreatic parenchymal phase that gradually decreases on portal and delayed phase on CT (arrow in **l, m, n**). Note dilatation of main pancreatic duct in the pancreas upstream to the tumor.

**Figure 2:**
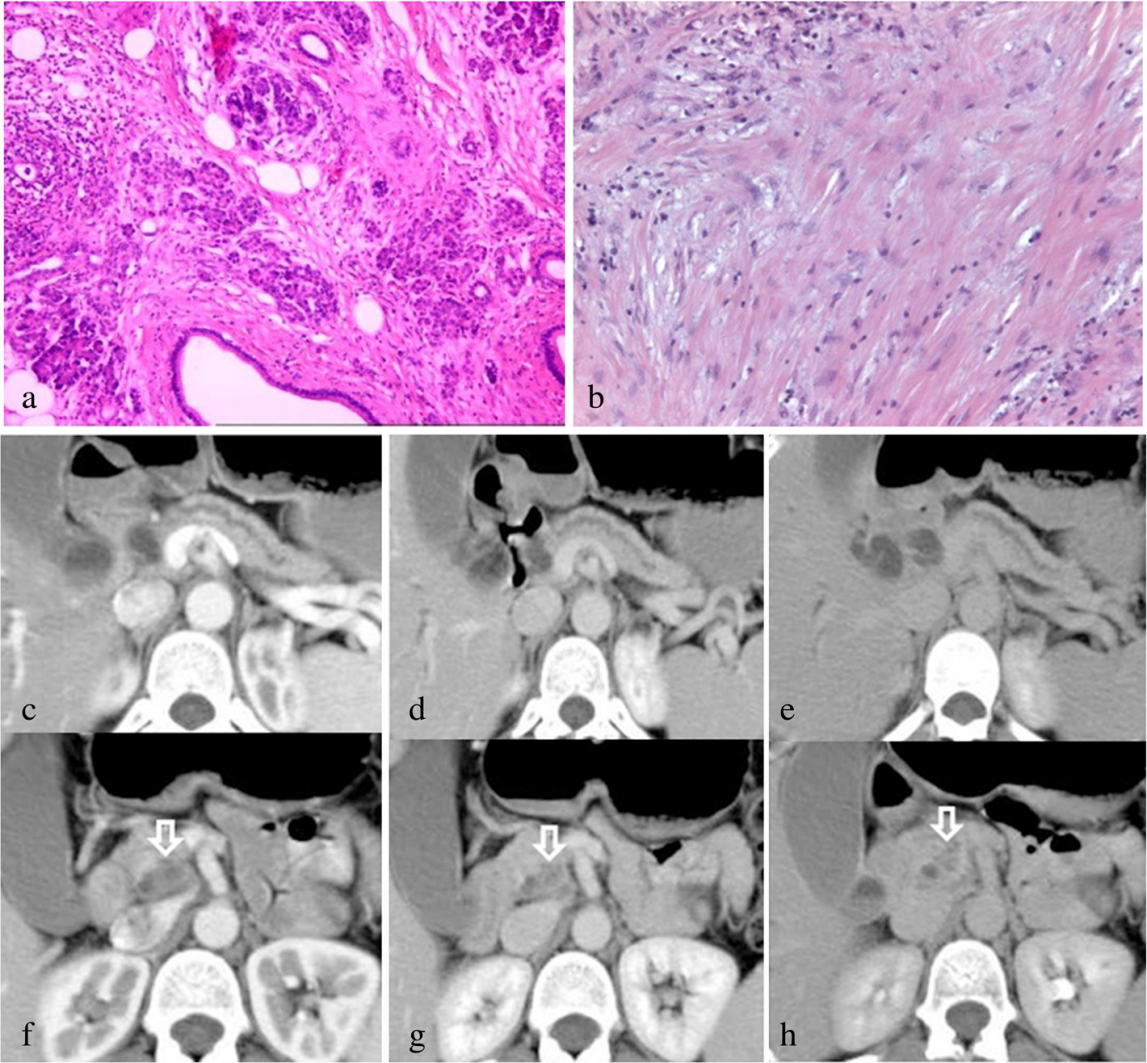
**Pancreatic ductal adenocarcinoma of the uncinate process of the pancreas: histology with triple-phase helical CT correlation in mild chronic pancreatitis of the pancreas upstream.** Extensive mild fibrosis in the upstream pancreatic tissue (HδE 100×) **(a)** and pancreatic ductal adenocarcinoma with severe fibrous stromal reaction (HδE 100×) **(b)**. CT shows maximum enhancement on portal venous phase for upstream pancreatic tissue **(c-e)** and increasing pattern for pancreatic ductal adenocarcinoma **(f-h)**. Note dilatation of main pancreatic duct in the pancreas upstream to the tumor.

A representative case of severe chronic pancreatitis in pancreas upstream to PDA and mild chronic pancreatitis of the downstream pancreas and mild chronic pancreatitis in pancreas upstream to PDA are reported in Figures 1 and 2 respectively.

## Discussion

Despite the recent advances in imaging and treatment, pancreatic ductal adenocarcinoma (PDA) continues to be a lethal disease. Recently, some studies reported PDA so called “isodense” on CT in 5–45% of cases and emphasize the ancillary sign (eg. main pancreatic duct dilatation or the interrupted duct sign) in the detection of these tumors [4,5,7,8].

The misdiagnosis owing to obscurity of a tumor on CT scan is related to the histopathological characteristics of the tumor [8,10,11] and surrounding pancreatic parenchyma [5].

CT enhancement of the PDA and surrounding pancreatic parenchyma to the tumor is correlated to the their degree of fibrosis. PDA with predominant fibrous component, such as intrahepatic cholangiocarcinoma, shows retention of contrast material [12-16]. A similar degree of fibrosis in the tumor and surrounding pancreatic parenchyma may determine an overlapping enhancement on MDCT that may preclude the detection of PDA, especially for small (≤ 2 cm) lesions.

Normal pancreatic parenchyma and mild chronic pancreatitis of pancreas downstream to PDA is related to regular and partial obstruction of drainage of this segment into the main duct through the accessory one respectively.

Main duct obstruction leads to a framework of obstructive chronic pancreatitis characterized by ductal dilatation and fibrous replacement of variable degree (mild or severe) of parenchyma upstream [10,11].

Few studies emphasize the usefulness of triphasic helical CT to differentiate the enhancement of pancreatic parenchyma and PDA [17]. Recently, Ishigami et al. [18], using triphasic MDCT technique, based on the retention of contrast material due to the fibrotic-stromal component of PDA, emphasized the usefulness of DP at 5 minutes for an optimal detection of small PDA visually isoattenuating on PPP. Moreover, a lot of articles regarding the use of CT perfusion for detection of PDA are available in recent literature [19]. However, although computed tomographic perfusion (CTp) imaging is a promising technique that allows functional imaging, as an adjunct to a morphologic CT examination, the measurements obtained with the CT perfusion software and their upgrades are not currently consistent and reproducible.

Our data show that type 1 pattern (normal pancreas) was observed only in downstream pancreas: this means that when pancreatic parenchyma downstream to the tumor is evaluable, the difference in attenuation between this one and the tumor allows the detection of PDA in all cases (38/38 patients; sensitivity of 100%). Otherwise, a coexisting pancreatitis in the pancreatic parenchyma upstream to the tumor, a very frequent condition, makes diagnosis difficult because of the overlapping of the attenuation values between the tumor and pancreas upstream.

According to our experience, small PDAs were isodense to the pancreas upstream to the tumor, and therefore unrecognizable, in 8 cases (8/38; 21%) at qualitative analysis and in 4 cases (4/38; 10,5%) at quantitative analysis; on DP two PDAs not detected on PPP and PVP were revealed.

The quantitative analysis at triphasic MDCT increases tumor detection with respect to visual analysis, showing a higher sensitivity in all phases, even for small PDAs isodense to the pancreatic parenchyma upstream to the tumor.

Our study has some limitations:

1) the retrospective nature of the study including a limited (38/127) selected patients with histologically proved PDA in which the relationship between enhancement and histopathology was obtained;

2) the characteristics of patients enrolled in the study, which were all surgically treated patients for a small PDA detected at MDCT, not including patients with an hypothetical misdiagnosis;

3) the DP at 5 minutes increases the radiation dose to the patient. Although recently has been proposed split-bolus CT protocol of the pancreas to reduce dose radiation, the DP is useful in the detection of visually isoattenuating lesions on PPP or on PVP [17,20-22] establishing the presence of obstructive chronic pancreatitis in the surrounding pancreas to the tumor.

## Conclusions

In conclusion the knowledge of the histologic features and enhancement patterns of PDA and surrounding pancreas at CT could be essential for improve research on methods to detect isoattenuating tumor. Our preliminary study shows that the quantitative analysis of the enhancement for PDA and surrounding parenchyma may be considered in order to increase the sensitivity of CT in the detection of small PDA.

## Competing interests

The authors declare that they have no competing interests.

## Authors’ contributions

MS, performed experiments, analyzed data, interpreted results of experiments, prepared figures, drafted manuscript, conception and design of research, approved final version of manuscript. FDS, TP, IP, MM, performed experiments, analyzed data, interpreted results of experiments, prepared figures, drafted manuscript. LP, ADA performed experiments, analyzed data, interpreted results of experiments, prepared figures, drafted manuscript, edited and revised manuscript. LC, LB interpreted results of experiments, edited and revised manuscript. AR conception and design of research, interpreted results of experiments, approved final version of manuscript. All authors read and approved the final manuscript.

## Pre-publication history

The pre-publication history for this paper can be accessed here:

http://www.biomedcentral.com/1471-230X/14/16/prepub

## Supplementary Material

Additional file 1 **Time-density-curves of tumor, pancreas upstream and pancreas downstream to the tumor at triphasic CT.** Time-density-curves show the tumor with a progressive enhancement throughout the three phases with maximum peak in DP; pancreatic parenchyma upstream to the tumor shows maximum enhancement in PVP that gradually decreases in DP; pancreatic parenchyma downstream to the tumor shows maximum enhancement peak during PPP followed by a rapid decline on PVP and DP. The mean attenuation values (HU ± SD) of pancreas upstream to the tumor were significantly higher than those of PDA on PPP, PVP and DP (p < 0.05) whereas the mean attenuation values of parenchyma downstream to the tumor were significantly higher than those of tumor in PPP and PVP (p < 0.05) but not significantly different in DP (p > 0.05). Pre-C: pre-contrast; PPP: pancreatic parenchymal phase; PVP = portal venous phase; DP = delayed phase. Click here for file

Additional file 2 **Comparison of difference in mean attenuation (HU) between tumor and pancreas upstream and downstream to the tumor at triphasic CT.** The dashed lines indicate the minimum difference of enhancement (10 HU) to detect the tumor respect to the surrounding pancreatic parenchyma. Pre-C: pre-contrast; PPP: pancreatic parenchymal phase; PVP = portal venous phase; DP = delayed phase. p-Up: pancreas upstream to the tumor; p-Down: pancreas downstream to the tumor. Click here for file
