# Supplementary material for: Detection of small (≤ 2 cm) pancreatic adenocarcinoma and surrounding parenchyma: correlations between enhancement patterns at triphasic MDCT and histologic features
Source: BMC Gastroenterol. 2014 Jan 21;14:16. doi: 10.1186/1471-230X-14-16 (PMC3903041; doi:10.1186/1471-230X-14-16)
Supplement: Additional file 1 — Time-density-curves of tumor, pancreas upstream and pancreas downstream to the tumor at triphasic CT. Time-density-curves show the tumor with a progressive enhancement throughout the three phases with maximum peak in DP; pancreatic parenchyma upstream to the tumor shows maximum enhancement in PVP that gradually decreases in DP; pancreatic parenchyma downstream to the tumor shows maximum enhancement peak during PPP followed by a rapid decline on PVP and DP. The mean attenuation values (HU ± SD) of pancreas upstream to the tumor were significantly higher than those of PDA on PPP, PVP and DP (p < 0.05) whereas the mean attenuation values of parenchyma downstream to the tumor were significantly higher than those of tumor in PPP and PVP (p < 0.05) but not significantly different in DP (p > 0.05). Pre-C: pre-contrast; PPP: pancreatic parenchymal phase; PVP = portal venous phase; DP = delayed phase. [file 1471-230X-14-16-S1.docx]

Additional file 1. **Time-density-curves of tumor, pancreas upstream and pancreas downstream to the tumor at triphasic CT**


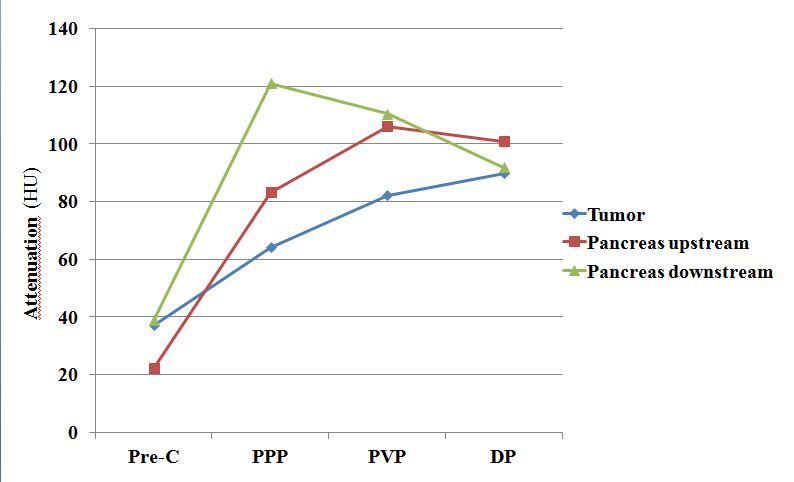


**Diagram 1 —**Time-density-curves show the tumor with a progressive enhancement throughout the three phases with maximum peak in DP; pancreatic parenchyma upstream to the tumor shows maximum enhancement in PVP that gradually decreases in DP; pancreatic parenchyma downstream to the tumor shows maximum enhancement peak during PPP followed by a rapid decline on PVP and DP.

The mean attenuation values (HU ± SD) of pancreas upstream to the tumor were significantly higher than those of PDA on PPP, PVP and DP (p < 0.05) whereas the mean attenuation values of parenchyma downstream to the tumor were significantly higher than those of tumor in PPP and PVP (p < 0.05) but not significantly different in DP (p > 0.05).

Pre-C: pre-contrast; PPP: pancreatic parenchymal phase; PVP= portal venous phase; DP=delayed phase.
