# Supplementary material for: Detection of small (≤ 2 cm) pancreatic adenocarcinoma and surrounding parenchyma: correlations between enhancement patterns at triphasic MDCT and histologic features
Source: BMC Gastroenterol. 2014 Jan 21;14:16. doi: 10.1186/1471-230X-14-16 (PMC3903041; doi:10.1186/1471-230X-14-16)
Supplement: Additional file 2 — Comparison of difference in mean attenuation (HU) between tumor and pancreas upstream and downstream to the tumor at triphasic CT. The dashed lines indicate the minimum difference of enhancement (10 HU) to detect the tumor respect to the surrounding pancreatic parenchyma. Pre-C: pre-contrast; PPP: pancreatic parenchymal phase; PVP = portal venous phase; DP = delayed phase. p-Up: pancreas upstream to the tumor; p-Down: pancreas downstream to the tumor. [file 1471-230X-14-16-S2.docx]

Additional file 2. **Comparison of difference in mean attenuation (HU) between tumor and pancreas upstream and downstream to the tumor at triphasic CT.**
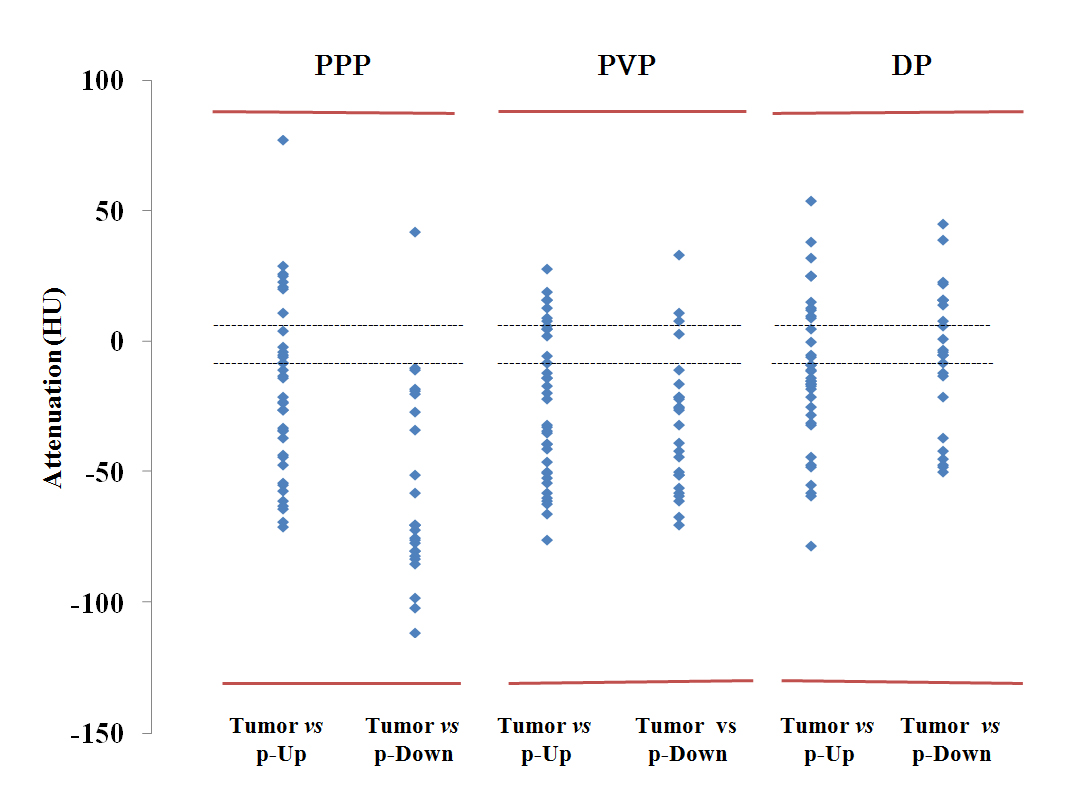


The dashed lines indicate the minimum difference of enhancement (10 HU) to detect the tumor respect to the surrounding pancreatic parenchyma. Pre-C: pre-contrast; PPP: pancreatic parenchymal phase; PVP= portal venous phase; DP=delayed phase. p-Up: pancreas upstream to the tumor; p-Down: pancreas downstream to the tumor.
